# DNA repair gene *ERCC2 *polymorphisms and associations with breast and ovarian cancer risk

**DOI:** 10.1186/1476-4598-7-36

**Published:** 2008-05-02

**Authors:** Dominique Bernard-Gallon, Rémy Bosviel, Laetitia Delort, Luc Fontana, Alain Chamoux, Nadège Rabiau, Fabrice Kwiatkowski, Nasséra Chalabi, Samir Satih, Yves-Jean Bignon

**Affiliations:** 1 Département d'Oncogénétique du Centre Jean Perrin, EA 2416 CBRV, 28 Place Henri Dunant, B.P. 38, 63001 Clermont-Ferrand Cedex 01, France; 2 CRNH, 58 rue Montalembert, 63009 Clermont-Ferrand Cedex 01, France; 3 Univ Clermont 1, UFR Médecine, Institut de Médecine du Travail, 28 place Henri Dunant, 63001 Clermont-Ferrand, France; 4 CHU Clermont-Ferrand, Service Santé Travail Environnement, 28 place Henri Dunant, 63001 Clermont-Ferrand cedex, France

## Abstract

Breast and ovarian cancers increased in the last decades. Except rare cases with a genetic predisposition and high penetrance, these pathologies are viewed as a polygenic disease. In this concept, association studies look for genetic variations such as polymorphisms in low penetrance genes, *i.e*. genes in interaction with environmental factors. DNA repair systems that protect the genome from deleterious endogenous and exogenous damages have been shown to have significantly reduced. In particular, enzymes of the nucleotide excision repair pathway are suspected to be implicated in cancer. In this study, 2 functional polymorphisms in a DNA repair gene *ERCC2 *were analyzed. The population included 911 breast cancer cases, 51 ovarian cancer cases and 1000 controls. The genotyping of 2 SNP (Single Nucleotide Polymorphism) was carried out on the population with the MGB (Minor Groove Binder) probe technique which consists of the use of the allelic discrimination with the Taqman^® ^method. This study enabled us to show an increase in risk of breast cancer with no oral contraceptive users and with women exhibiting a waist-to-hip ratio (WHR) > 0.85 for Asn homozygous for *ERCC2 312*.

## Background

High levels of DNA damage, caused by excessive exposure to carcinogens, might be responsible for increased breast cancer susceptibility in women known to have significantly reduced DNA repair proficiencies [1]. In particular, dysfunctions of the nucleotide excision repair (NER) pathway are known or suspected to be involved in cancer. The DNA helicase encoded by the excision repair cross-complementing group 2 gene *ERCC2 *(formely *XPD*) is one of seven nucleotide excision repair enzymes that cause *Xeroderma Pigmentosum *when mutated in germ line [2]. Several polymorphisms have been identified in this gene and particularly the *Asp312Asn ERCC2 *polymorphism which consists of the substitution of a G to A resulting in an amino acid change in the coding region, and the *Lys751Gln *which consists in a A to C substitution in the coding region [3]. A change of amino acid is able to modify the effect of protein more or less, which can translate by an effect on the systems of repair and consequently on the carcinogenesis. Conflicting data on the roles of these polymorphisms on cancer risk including breast and ovarian cancers have been described [4-6].

The objective of this study was to establish the role of two functional polymorphisms of a DNA repair gene *ERCC2 *in the risk of breast or ovarian cancer. We investigated the possible interactions between these polymorphisms and specific environmental factors (reproductive factors, body mass index, tobacco smoking...) which could influence the risk of cancer.

## Results

### Risk associated with individual SNPs

In this breast cancer population (Table 1), no significant differences were found between breast cancer cases and controls. A trend to the increase in breast cancer risk could be observed with heterozygous women for the SNP at position 312 of ERCC2 protein (OR = 1.06; 95% CI = 0.93–1.21) after adjustment for age. For ovarian cancer (Table 2), there was no significant modification in the risk for the two studied SNP.

**Table 1:** *ERCC2 Asp312Asn *and *ERCC2 Lys751Gln *polymorphisms and breast cancer risk

**Genotype**	**Cases (%)**	**Controls (%)**	**OR (95%CI)**	ORadj* (95%CI)	***P *value**
ERCC2 Asp312Asn					
Asp/Asp	403 (45)	458 (46)	1.00 (reference)	1.00 (reference)	
Asp/Asn	383 (42)	418 (42)	1.04 (0.86–1.26)	1.06 (0.93–1.21)	
Asn/Asn	118 (13)	118 (12)	1.14 (0.85–1.52)	1.12 (0.86–1.46)	0.68
ERCC2 Lys751Gln					
Lys/Lys	121 (13)	119 (11)	1.00 (reference)	1.00	
Lys/Gln	419 (46)	446 (44)	0.92 (0.69–1.23)	0.92 (0.80–1.05)	
Gln/Gln	368 (40)	430 (43)	0.84 (0.63–1.12)	0.84 (0.64–1.09)	0.43

* Adjusted for age

**Table 2:** *ERCC2 Asp312Asn *and *ERCC2 Lys751Gln *polymorphisms and ovarian cancer risk

**Genotype**	**Cases (%)**	**Controls (%)**	**OR (95%CI)**	ORadj* (95%CI)	***P *value**
ERCC2 Asp312Asn					
Asp/Asp	21 (41)	458 (46)	1.00 (reference)	1.00 (reference)	
Asp/Asn	28 (55)	418 (42)	1.46 (0.82–2.60)	0.95 (0.62–1.45)	
Asn/Asn	2 (4)	118 (12)	0.37 (0.09–1.51)	0.9 (0.39–2.12)	0.088

ERCC2 Lys751Gln					
Lys/Lys	1 (2)	119 (12)	1.00 (reference)	1.00 (reference)	
Lys/Gln	31 (61)	446 (45)	8.27 (1.54–44.43)	1.08 (0.70–1.66)	
Gln/Gln	19 (37)	430 (43)	5.26 (0.86–32.18)	1.16 (0.49–2.75)	0.025

* Adjusted for age

### Interaction between genetic factors and anthropometric/lifestyle factors

Results concerning interactions between environmental factors and risk of breast cancer were reported in Table 3. Nonsmoker homozygous Asn at position 312 of *ERCC2 *tended towards an increase in the risk of developing breast cancer (OR = 1.4; 95% CI = 0.98–2.02). Heterozygous individuals for *ERCC2 Asp312Asn *which received a hormonal replacement therapy (HRT) exhibited a risk to develop breast cancer (OR = 1.39; 95% CI = 0.97–1.99). A significant increase in the risk was observed for individuals who did not take oral contraceptives and who were homozygous Asn at position 312 of *ERCC2 *(OR = 1.66; 95% CI = 1.08–2.53). An increase was observed for heterozygous individuals at position 751 of *ERCC2 *who took oral contraceptives (OR = 1.54; 95% CI = 1.00–2.36). When age at first oral contraceptive use was after 23 years, a significant increase in breast cancer risk was obtained for heterozygous *ERCC2 Lys751Gln *(OR = 2.22; 95% CI = 1.15–4.29). Lastly, women with a waist-to-hip ratio (WHR) > 0.85 and homozygous for position 312 of *ERCC2 *exhibited a significant increase in the risk of breast cancer (OR = 1.96; 95% CI = 1.12–3.43).

**Table 3:** *ERCC2 Asp312Asn*, *ERCC2 Lys751Gln *polymorphisms and potential breast cancer risk factors

	**Nonsmokers**				**Smokers**			
Genotype	Cases (%)	Controls (%)	OR (CI 95%)	*P *value	Cases (%)	Controls (%)	OR (CI 95%)	*P *value

*ERCC2 Asp312Asn*								
*Asp/Asp*	283 (45)	299 (47)	1.00 (reference)		102 (42)	159 (44)	1.00 (reference)	
*Asp/Asn*	258 (41)	270 (43)	1.01 (0.80–1.28)		111 (46)	146 (41)	1.19 (0.83–1.68)	
*Asn/Asn*	85 (14)	64 (10)	1.4 (0.98–2.02)	0.16	29 (12)	54 (15)	0.84 (0.50–1.40)	0.36

	No HRT* users				**HRT users**			

Genotype	Cases (%)	Controls (%)	OR (CI 95%)	*P *value	Cases (%)	Controls (%)	OR (CI 95%)	*P *value

*ERCC2 Asp312Asn*								
*Asp/Asp*	175 (46)	137 (48)	1.00 (reference)		75 (40)	216 (47)	1.00 (reference)	
*Asp/Asn*	152 (40)	119 (41)	1.00 (0.72–1.39)		92 (49)	191 (42)	1.39 (0.97–1.99)	
*Asn/Asn*	54 (14)	31 (11)	1.36 (0.83–2.23)	0.44	19 (10)	50 (11)	1.09 (0.61–1.97)	0.20

	No OC** users				**OC users**			

Genotype	Cases (%)	Controls (%)	OR (CI 95%)	*P *value	Cases (%)	Controls (%)	OR (CI 95%)	*P *value

*ERCC2 Asp312Asn*								
*Asp/Asp*	240 (46)	215 (50)	1.00 (reference)		143 (42)	243 (43)	1.00 (reference)	
*Asp/Asn*	208 (40)	176 (41)	1.06 (0.81–1.39)		161 (47)	242 (43)	1.13 (0.85–1.51)	
*Asn/Asn*	74 (14)	40 (9)	1.66 (1.08–2.53)	0.061	40 (12)	78 (14)	0.87 (0.56–1.34)	0.44

*ERCC2 Lys751Gln*								
*Lys/Lys*	79 (15)	39 (9)	1.00 (reference)		38 (11)	80 (14)	1.00 (reference)	
*Lys/Gln*	223 (43)	198 (46)	0.56 (0.36–0.85)		181 (52)	248 (44)	1.54 (1.00–2.36)	
*Gln/Gln*	222 (42)	195 (45)	0.56 (0.37–0.86)	0.018	127 (37)	235 (42)	1.14 (0.73–1.77)	0.044

	**Age at first OC use ≤ 23 years**				**Age at first OC > 23 years**			

Genotype	Cases (%)	Controls (%)	OR (CI 95%)	*P *value	Cases (%)	Controls (%)	OR (CI 95%)	*P *value

*ERCC2 Lys751Gln*								
*Lys/Lys*	23 (14)	46 (15)	1.00 (reference)		15 (8)	34 (13)	1.00 (reference)	
*Lys/Gln*	80 (50)	145 (47)	1.10 (0.62–1.95)		101 (55)	103 (40)	2.22 (1.15–4.29)	
*Gln/Gln*	58 (36)	115 (38)	1.01 (0.56–1.82)	0.89	69 (37)	120 (47)	1.3 (0.66–2.56)	0.008

	**Waist-to-hip ratio = 0.85**				**Waist-to-hip ratio > 0.85**			

Genotype	Cases (%)	Controls (%)	OR (CI 95%)	*P *value	Cases (%)	Controls (%)	OR (CI 95%)	*P *value

*ERCC2 Asp312Asn*								
*Asp/Asp*	252 (46)	356 (46)	1.00 (reference)		151 (42)	102 (48)	1.00 (reference)	
*Asp/Asn*	234 (43)	326 (42)	1.01 (0.80–1.28)		149 (42)	92 (43)	1.09 (0.76–1.57)	
*Asn/Asn*	60 (11)	98 (13)	0.86 (0.60–1.24)	0.68	58 (16)	20 (9)	1.96 (1.12–3.43)	0.06

* HRT = Hormone Replacement Therapy

** OC = Oral Contraceptive

Concerning ovarian cancer (Table 4), a significant increase in the risk was found for heterozygous *ERCC2 Lys751Gln *and who had menarche before 13 years old (OR = 6.03; 95% CI = 1.02–35.78). Heterozygous individuals *ERCC2 Asp312Asn *who did not take oral contraceptives had a significant increase in the risk of ovarian cancer (OR = 2.16; 95% CI = 1.08–4.33). Heterozygous *ERCC2 Asp312Asn *with BMI > 25 exhibited a significant increase in ovarian cancer risk (OR = 3.5; 95% CI = 1.00–12.26), while heterozygous *ERCC2 Lys751Gln *with body mass index (BMI) < 25 exhibited an upward tendency in ovarian cancer risk (OR = 5.64; 95% CI = 0.93–33.99). Heterozygous *ERCC2 Lys751Gln *with WHR = 0.85 also exhibited an upward tendency in the risk of ovarian cancer (OR = 5.68; 95% CI = 0.94–34.25).

**Table 4:** *ERCC2 Asp312Asn*, *ERCC2 Lys751Gln *polymorphisms and potential ovarian cancer risk factors

	**Age at menarche = 13 years**				**Age at menarche > 13 years**			
Genotype	Cases (%)	Controls (%)	OR (CI 95%)	*P *value	Cases (%)	Controls (%)	OR (CI 95%)	*P *value

*ERCC2 Lys751Gln*								
*Lys/Lys*	1 (3)	87 (13)	1.00 (reference)		0 (0)	32 (10)	-	
*Lys/Gln*	21 (57)	303 (46)	6.03 (1.02–35.78)		10 (71)	175 (52)	1.00 (reference)	
*Gln/Gln*	15 (41)	271 (41)	4.82 (0.6–30.62)	0.14	4 (29)	127 (38)	0.55 (0.17–1.77)	0.32

	No OC* users				**OC users**			

Genotype	Cases (%)	Controls (%)	OR (CI 95%)		Cases (%)	Controls (%)	OR (CI 95%)	

*ERCC2 Asp312Asn*								
*Asp/Asp*	13 (35)	215 (50)	1.00 (reference)		5 (50)	243 (43)	1.00 (reference)	
*Asp/Asn*	23 (62)	176 (41)	2.16 (1.08–4.33)		5 (50)	242 (43)	1.00 (0.29–3.51)	
*Asn/Asn*	1 (3)	40 (9)	0.41 (0.06–3.05)	0.032	0 (0)	78 (14)	-	0.45

*ERCC2 Lys751Gln*								
*Lys/Lys*	0 (0)	39 (9)	-		0 (0)	80 (14)	-	
*Lys/Gln*	27 (73)	198 (46)	1.00 (reference)		4 (40)	248 (44)	1.00 (reference)	
*Gln/Gln*	10 (27)	195 (45)	0.38 (0.18–0.78)	0.0085	6 (60)	235 (42)	1.58 (0.45–5.62)	0.7**

	**BMI ≤ 25**				**BMI >25**			

Genotype	Cases (%)	Controls (%)	OR (CI 95%)		Cases (%)	Controls (%)	OR (CI 95%)	

*ERCC2 Asp312Asn*								
*Asp/Asp*	18 (47)	311 (45)	1.00 (reference)		3 (23)	147 (48)	1.00 (reference)	
*Asp/Asn*	19 (50)	292 (43)	1.12 (0.58–2.18)		9 (69)	126 (41)	3.5 (1.00–12.26)	
*Asn/Asn*	1 (3)	84 (12)	0.21 (0.03–1.29)	0.19	1 (8)	34 (11)	1.44 (0.15–14.11)	0.13

*ERCC2 Lys751Gln*								
*Lys/Lys*	1 (3)	84 (12)	1.00 (reference)		0 (0)	35 (11)	-	
*Lys/Gln*	21 (55)	313 (45)	5.64 (0.93–33.99)		10 (77)	133 (43)	1.00 (reference)	
*Gln/Gln*	16 (42)	291 (42)	4.62 (0.72–29.53)	0.17	3 (23)	139 (45)	0.29 0.08–0.99)	0.048

	**Waist-to-hip ratio = 0.85**				**Waist-to-hip ratio > 0.85**			

Genotype	Cases (%)	Controls (%)	OR (CI 95%)		Cases (%)	Controls (%)	OR (CI 95%)	

*ERCC2 Lys751Gln*								
*Lys/Lys*	1 (4)	102 (13)	1.00 (reference)		0 (0)	17 (8)	-	
*Lys/Gln*	19 (70)	341 (44)	5.68 (0.94–34.25)		12 (50)	105 (49)	1.00 (reference)	
*Gln/Gln*	7 (26)	338 (43)	2.11 (0.27–16.57)	0.02	12 (50)	92 (43)	1.14 (0.49–2.66)	0.76

* OC = Oral Contraceptive

** after Yates correction

## Discussion

Nucleotide Excision Repair (NER) pathway is a key DNA repair system. A dysfunction of this system would result in higher cancer susceptibility because individuals would be more exposed to carcinogens. Exogenous (cigarette smoke, pollutants) and endogenous carcinogens are potential breast and ovarian cancer risk factors. Above all, exposition to estrogens seemed to be the major risk factor. Indeed, estrogens had proliferative effects on mammary cells and could be metabolized in potential carcinogens and induce DNA damage [7]. Many investigations were done concerning breast cancer risk and DNA repair polymorphisms. At the opposite, few studies were done with ovarian cancer which exhibited a lower incidence but a high mortality rate. So we studied two polymorphisms in *ERCC2 *DNA repair gene because these polymorphisms might modify the exposure of women to estrogens.

An early age at menarche increases the risk of ovarian and breast cancers, probably due to the prolonged exposure of breast epithelium to endogenous hormones. Similarly a late age at menopause is considered as a risk for cancer because it increases the number of ovulatory cycles [8]. Concerning parity, a dual effect of pregnancy is reported. On one hand, parity inhibits the early stages of mammary carcinogenesis (long-term risk reduction) due to the terminal differentiation of breast tissue. On the other hand, there is a short-term risk increase because of the proliferation of breast tissue in response to high gestational hormone levels which render the mammary gland more susceptible to carcinogens [9]. Overall early pregnancy and high parity are considered to have a protective effect [10-13].

Oral contraceptive use was by far the most influential factor on breast cancer development. However, the hormonal replacement therapy generates a prolonged exposure to estrogens during life, leading to an increased risk of breast and ovarian cancers. At the opposite, the established protective effect of OC use in ovarian cancer may also be due to the suppression of the LH peak and to a decrease of endogenous estradiol production. If estrogens are related to an increased risk of ovarian cancer, oral contraception might have some protective effect by lowering the overall level of estrogens [14,15].

Studies of the relationship between BMI and the risk of ovarian cancer have been inconclusive, finding either a positive correlation [16-23], no relationship [24-31], or negative association [32]. Our results suggested an interaction with menopausal status, with higher BMI being more associated with ovarian cancer risk in premenopausal women than in postmenopausal women, in agreement with Beehler et al (2006) [33]. Greer et al (2005) showed a slight increase in risk with weight during adulthood and later in life, which was most apparent among nulliparous women [34]. Fairfield et al (2002) did not find any association between recent BMI and risk of ovarian cancer, but reported that a high BMI during early adulthood was associated with an increased risk of premenopausal cancer [26]. A case-control study reported an association between increased BMI at age 18 and during most adult life and ovarian cancer [23].

We found a strong relationship between waist-to-hip ratio and the risk of ovarian cancer regardless of menopausal status, underscoring the role of central adiposity in the induction of ovarian cancer [35].

In addition, we studied tobacco smoking, the role of which was very important in ovarian and breast cancers. For ovarian and breast cancers, the role of the tobacco is complex. Some of cigaret smoke compounds will have an anti-estrogen effect, while others such as HAP, AAH and N-nitrosamines will act as carcinogens by acting directly on DNA. The lipophilic HAP are stored in fat tissues, which include the mammary glands and tissues surrounding the ovaries [7].

Our results showed an increase in the risk of breast cancer in individual heterozygous for *ERCC2 Asp312Asn *with women receiving a menopause substitutive treatment and an increased risk for ovarian cancer with no oral contraceptive uses, and/or BMI > 25. For heterozygous *ERCC2 Lys751Gln*, an increased risk for breast cancer was found with age at first OC use after 23 years. Heterozygous *ERCC2 Lys751Gln *exhibited a higher risk for ovarian cancer when age at menarche was before 13 years, BMI < 25 and WHR < 0.85.

Besides, homozygous *Asn *for *ERCC2 312 *tended to have an increase in breast cancer risk among nonsmokers, and a significant increase in breast cancer risk among OC users or with WHR > 0.85. It might be a combined effect between DNA damage caused by estrogens and reduced DNA repair proficiency with *ERCC2*. For the other first results, the explanation might be a reduced DNA repair proficiency with *ERCC2 *but it was found when the interaction between genetic factors and anthropometric/lifestyle factors was considered.

Contradictory results were found in the literature. Tang et al (2002) did not show an association between these two SNP in *ERCC2 *and breast cancer risk whereas a lower A allele frequency in *ERCC2 Asp312Asn* was found in a Chinese population by Zhang et al (2005) [5,36]. On the contrary, a case-control study in Portugal revealed that women with the *Asn312Asn *or the *Gln751Gln *genotype presented a three-fold risk of ovarian cancer in comparison with heterozygous or wild-type homozygous women [3].

## Conclusion

This study enabled us to show an increase in risk of breast cancer with no oral contraceptive users and with women exhibiting a waist-to-hip ratio superior to 0.85 for Asn homozygous for *ERCC2 312*.

## Subjects and Methods

### Study Subjects

911 breast cancer cases and 51 ovarian cancer cases (aged 26–89 years), from the Auvergne region in France were included. They are belonging to the COSA program (Breast and Ovarian Cancer in the Auvergne region) that consisted of the recruitment of identified women from different clinics and hospitals in the Auvergne region in France from November 1996 through November 1999. Eligible subjects were women who had been diagnosed with breast or ovarian cancer with no *BRCA *mutation and no more than one first degree relative breast cancer.

1,000 healthy control women (aged 24–85 years) were recruited in a mammographic screening center from July 2005 to April 2006. Eligible controls were women with no previous history of cancer and resident in the Auvergne region. All study subjects received counseling and provided written consent for the study.

### Data collection

Participants completed a questionnaire about their medical and reproductive histories in particular. Breast and ovarian cases filled in the questionnaire at the time of clinic appointment whereas controls were interviewed at the medical center at the time of enrollment. Data regarding reproductive history (including gravidity, parity, age at first-full term pregnancy, history of breastfeeding, age at menarche and menopause, menstrual cycle regularity, hormone replacement therapy), use of oral contraceptives (OC) ("Ever use" of OC was defined as at least 3 months of use), age at first OC use and duration of OC, anthropometric characteristics (height, weight, weight at age 20, waist and hip measurements), smoking status ("ever smoker" was defined as at least 1 year of smoking) were collected.

### Genotyping

For breast and ovarian cancer patients and healthy women, genomic DNAs were extracted from whole blood using DNA extraction kit by Euromedex according to the manufacturer's protocol (Euromedex, Souffelweyersheim, France). Two functional polymorphisms of a DNA repair gene *ERCC2 *were selected (Table 5). The corresponding probes were ordered at the company Applied Biosystems. Allelic discrimination using fluorogenic probes (5' nuclease assay, Taqman^®^) was chosen for genotyping on the ABI PRISM 7700 Sequence Detection Systems (Applied Biosystems, Foster City, CA, USA) and consisted of the use of allele-specific fluorogenic probes [37]. Sixteen nanograms of DNA were amplified by AmpliTaq Gold DNA polymerase which was included in Taqman^® ^Universal Master Mix (Applied Biosystems, Foster City, CA, USA). The PCR reactions were as follows: one step of 10 min at 95°C followed by 40 cycles of two-step PCR with denaturation at 92°C for 15s and annealing and extension at 60°C for 1 min. Ten percent of all samples were genotyped again for quality control.

**Table 5:** Two functional polymorphisms were studied in a DNA repair gene (*ERCC2*)

**Gene Symbols**	**Names**	**Studied Polymorphisms (SNP)**	**Polymorphism NCBI References**
*ERCC2/XPD*	Excision Repair Cross-Complementing group 2	Asp312Asn [G23591A (exon 10)]	rs1799793
	Xeroderma Pigmentosum group D	Lys751Gln [A35931C (exon 23)]	rs13181

### Statistical Analysis

The software SEM (Centre Jean Perrin, Clermont Ferrand, France) was used for data analyses [38]. Standard descriptive statistics consisted in means plus standard deviation for quantitative data. Comparisons between cases and controls were performed using *Chi*^2^-squared test for qualitative parameters. Univariate and multivariate analyses were performed to determine cancer risk factors in our populations, and odds ratios (ORs) with corresponding 95% confidence interval (95% CI) were estimated.

Allelic frequencies and the distribution of genotype were compared within the two populations using *Chi*^2 ^analysis and ORs with 95% CI. Yates correction was performed when the number of cases was inferior to 5. The potential confounding effects of known breast cancer risk factors (age at menarche, number of children, age at first child birth, menopause, breastfeeding, oral contraceptive (OC) use, age at first OC use, body mass index [BMI], BMI at 20 years old, waist-to-hip ratio [WHR] and smoking) were evaluated by adjusting for unconditional logistic regression and calculation of ORs. As no changes in ORs were noted, results were reported without adjustment for these factors.

## Competing interests

The authors declare that they have no competing interests.

## Authors' contributions

DBG, LD was responsible for genotyping polymorphisms, statistical analyses and interpretation, and drafting of the manuscript; RB, NC, SS, NR contributed with the coordination of the study and helped in statistical analyses; FK provided expertise in data analyses; YJB, DBG, LF, AC contributed to the study design and manuscript preparation. All authors participated in the preparation of the manuscript and approved the final version.
